# Assessment of Baseline Plasma p‐tau217 in TANGO, a Randomized, Placebo‐controlled Phase 2 Study of Gosuranemab in Patients with Early Alzheimer’s Disease

**DOI:** 10.1002/alz.095703

**Published:** 2025-01-09

**Authors:** Julie Czerkowicz, Rong Zhang, Jessica Collins, Carrie E. Rubel, John O'Gorman, Melanie Shulman, Danielle Graham

**Affiliations:** ^1^ Biogen, Cambridge, MA USA; ^2^ Cytel Inc, Cambridge, MA USA

## Abstract

**Background:**

TANGO was a Phase 2 clinical study designed to assess the safety and efficacy of gosuranemab, an anti‐tau monoclonal antibody, in participants with mild cognitive impairment due to Alzheimer’s disease (AD) or with mild AD dementia. Despite robust target engagement of unbound N‐terminal tau, the clinical efficacy endpoint was not met. In this exploratory analysis of TANGO participants, we examine plasma p‐tau217 levels to assess the feasibility of using this biomarker to identify patients with AD pathology and predict disease progression.

**Methods:**

Plasma was collected from 554 randomized TANGO participants at baseline and up to Week 78. Plasma p‐tau217 was measured using the ALZpath Quanterix Simoa assay. Plasma p‐tau181 was measured using the Quanterix Simoa p‐tau181 V2 Advantage assay. Tau PET was measured in a sub‐study of 357 participants at baseline and Weeks 52 and 78 using ^18^F‐MK‐6240. Amyloid PET was measured at screening using ^18^F‐florbetapir in n = 322 participants Clinical decline was measured using the CDR‐SB, MMSE, ADAS‐Cog13 and ADCS‐ADL cognitive assessment scales. Statistical analysis was performed using Spearman correlation coefficient.

**Results:**

Plasma p‐tau217 and plasma p‐tau181 were correlated at baseline (Spearman = 0.7617, p‐value <0.0001). Baseline plasma p‐tau217 levels were correlated with both baseline amyloid PET using SUVR in a composite region of interest (Spearman = 0.4294, p‐value <0.0001) and baseline Tau PET using SUVR in composite regions of interest corresponding to Braak stages I‐II, III‐IV, and V‐VI (Spearman range = 0.4075‐0.6163, p = value <0.0001). Participants with higher concentrations of plasma ptau217 at baseline showed a greater rate of clinical decline at Week 78.

**Conclusions:**

The correlations of plasma p‐tau217 with amyloid and tau PET at baseline suggest a relationship with both underlying pathological hallmarks of AD. Similar to results from observational cohorts, correlation of baseline plasma p‐tau217 and clinical decline observed during the TANGO trial supports the utility of plasma p‐tau217 as a potential prognostic biomarker of disease. Data from additional studies are needed to define appropriate use cases and relevant thresholds for plasma p‐tau217 as a biomarker of disease pathology that can be used to prescreen patients in clinical trials.

